# Development and validation of a web-based dynamic nomogram to predict individualized risk of severe carotid artery stenosis based on digital subtract angiography

**DOI:** 10.3389/fneur.2025.1565395

**Published:** 2025-03-24

**Authors:** Jian Huang, Zhuoran Li, Xiaozhu Liu, Lirong Kuang, Shengxian Peng

**Affiliations:** ^1^Scientific Research Department, First People’s Hospital of Zigong City, Zigong, China; ^2^Department of Ultrasound, Sir Run Run Shaw Hospital, Zhejiang University College of Medicine, Hangzhou, China; ^3^Department of Neurology, Liuzhou Traditional Chinese Medical Hospital, The Third Affiliated Hospital of Guangxi University of Chinese, Liuzhou, China; ^4^Department of Emergency Medicine, Beijing Chaoyang Hospital, Capital Medical University, Beijing, China; ^5^Emergency and Critical Care Medical Center, Beijing Shijitan Hospital, Capital Medical University, Beijing, China; ^6^Department of Ophthalmology, Wuhan Wuchang Hospital, Wuchang Hospital Affiliated to Wuhan University of Science and Technology, Wuhan, China

**Keywords:** non-invasive, big data, web-based, individualized prediction model, carotid artery stenosis, ischemic stroke, risk factors

## Abstract

**Objectives:**

Delays in diagnosing severe carotid artery stenosis (CAS) are prevalent, particularly in low-income regions with limited access to imaging examinations. CAS is a major contributor to the recurrence and poor prognosis of ischemic stroke (IS). This retrospective cohort study proposed a non-invasive dynamic prediction model to identify potential high-risk severe carotid artery stenosis in patients with ischemic stroke.

**Methods:**

From July 2017 to March 2021, 739 patients with ischemic stroke were retrospectively recruited from the Department of Neurology at Liuzhou Traditional Chinese Medical Hospital. Risk factors for severe CAS were identified using the least absolute shrinkage and selection operator (LASSO) and multivariate logistic regression (MLR) methods. The model was constructed after evaluating multicollinearity. The model’s discrimination was assessed using the C-statistic and area under the curve (AUC). Its clinical utility was evaluated through the decision curve analysis (DCA) and the clinical impact curve (CIC). Calibration was examined using a calibration plot. To provide individualized predictions, a web-based tool was developed to estimate the risk of severe CAS.

**Results:**

Among the patients, 488 of 739 (66.0%) were diagnosed with severe CAS. Six variables were incorporated into the final model: history of stroke, serum sodium, hypersensitive C-reactive protein (hsCRP), C-reactive protein (CRP), basophil percentage, and mean corpuscular hemoglobin concentration (MCHC). Multicollinearity was ruled out through correlation plots, variance inflation factor (VIF) values, and tolerance values. The model demonstrated good discrimination, with a C-statistic/AUC of 0.70 in the test set. The DCA and CIC indicated that clinical decisions based on the model could benefit IS patients. The calibration plot showed strong concordance between predicted and observed probabilities. The web-based prediction model exhibited robust performance in estimating the risk of severe CAS.

**Conclusion:**

This study identified six key risk factors for severe CAS in IS patients. In addition, we developed a web-based dynamic nomogram to predict the individual risk of severe CAS. This tool can potentially support tailored, risk-based, and time-sensitive treatment strategies.

## Introduction

Ischemic stroke (IS) is a life-threatening condition and a major global public health concern. IS patients suffer from severe atherosclerotic stenosis, particularly carotid artery stenosis (CAS) (1). The poor prognosis of IS patients is often attributed to reduced cerebral blood flow caused by severe arterial stenosis. In addition, severe CAS is an independent risk factor for the early recurrence of IS, underscoring the importance of early diagnosis and prevention. Timely prediction and intervention for severe CAS can substantially improve IS outcomes (2). The severity of CAS also significantly influences treatment decisions and clinical outcomes. For instance, endovascular therapy is often recommended for patients with severe arterial stenosis or occlusion. Digital subtraction angiography (DSA) remains the gold standard for diagnosing CAS, but it is both invasive and costly (3). Currently, there are limited non-invasive tools specifically designed to assess severe CAS in IS patients who lack access to imaging examinations (4). Thus, developing a practical, non-invasive, and individualized prediction model for severe CAS in IS patients is imperative. Nomograms have been widely recognized for their accuracy in calculating the probability of clinical outcomes. The nomogram model accurately calculated the possibility of outcome (5, 6). Compared to traditional predictive models, web-based dynamic nomograms for CAS prediction have gained attention due to their superior predictive performance and user-friendly interfaces.

In this study, we retrospectively analyzed 739 IS patients using a big data intelligence platform. We identified risk factors for severe CAS using the least absolute shrinkage and selection operator (LASSO) and multivariate logistic regression (MLR) methods. After evaluating the multicollinearity of prognostic factors, we developed and internally validated a non-invasive, individualized prediction model for severe CAS. Finally, the web-based dynamic nomogram was made publicly accessible online, providing dynamic diagnostic information to guide individualized treatment strategies for IS patients.

## Methods

### Patient population

**Figure 1:**
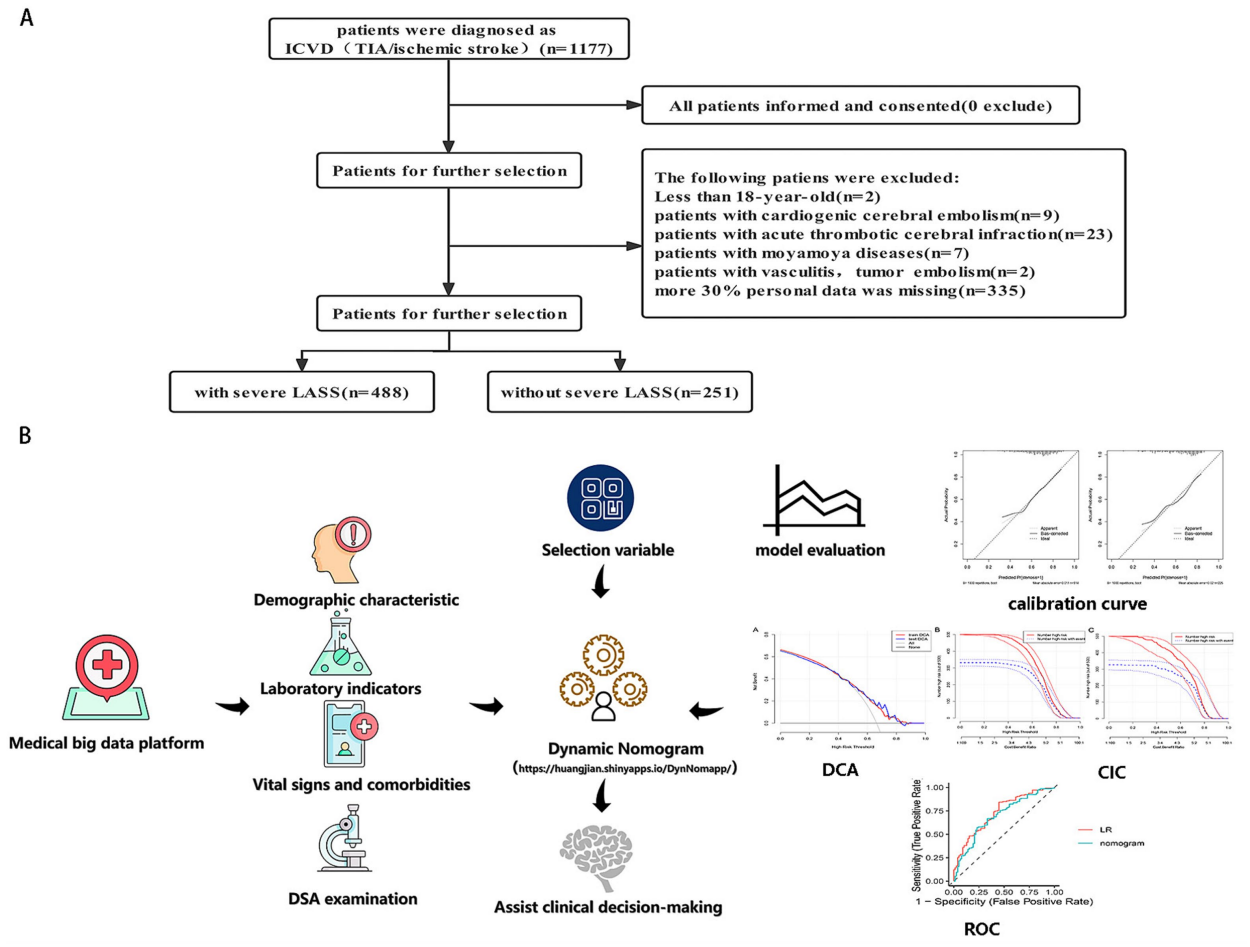
**(A)** The flowchart of all patients’ selection. **(B)** The conceptual framework of this study, including data collection, model development, and evaluation. LASSO, least absolute shrinkage and selection operator; CAS, carotid artery stenosis.

Between July 2017 and March 2021, 1,177 ischemic stroke (IS) patients were retrospectively recruited from the Department of Neurology, Liuzhou Traditional Chinese Medical Hospital. All patients underwent digital subtraction angiography (DSA) for the evaluation of carotid artery stenosis (CAS), assessed by two neurologists using the North American Symptomatic Carotid Endarterectomy Trial (NASCET) method. Patients meeting any of the following criteria were excluded: (1) less than 18 years old; (2) patient with acute cardiogenic cerebral embolism or acute thrombotic cerebral infarction; (3) patients with vasculitis, moyamoya disease, abnormal coagulation, and tumor embolism (7); and (4) patient with more than 30% of personal data missing. Ultimately, 739 IS patients were included in the analysis. The patient selection flowchart is shown in Figure 1. This retrospective study was conducted using anonymized data and was approved by the Ethics Review Board of Liuzhou Traditional Chinese Medical Hospital, with a waiver for informed consent.

### Data imputation and primary outcome

Missing data were imputed using the k-nearest neighbor (KNN) algorithm. Patients were randomly divided into a training set (*n* = 514) and a test set (*n* = 225) using a 7:3 ratio. The primary outcome was defined as severe CAS (stenosis ≥70%) based on the result of DSA.

### Predictor variables

Clinical data were collected for each participant based on clinical expertise and prior studies on similar topics (8). Baseline characteristics, including demographic information, comorbidities, and laboratory test results, were recorded within 24 h of initial hospital admission.

### Statistical analysis

All statistical analyses were performed using R software (version 4.1.3) and RStudio (version 1.1.456). Baseline characteristics between groups were compared using the “CBCgrps” and “nortest” R packages. Predictive factors were selected using the LASSO method with the “glmnet” package. Multicollinearity among variables was assessed using the “corrplot” and “cra” packages. Model development and calibration were conducted using the “rms” and “regplot” packages. The clinical usefulness of the non-invasive prediction model was assessed through decision curve analysis (DCA) and clinical impact curve (CIC) plots generated using the “rmda” package. LASSO regression was used to reduce data dimensionality (9), while MLR was applied to identify independent risk factors for severe CAS. A web-based dynamic nomogram was constructed using the “DynNom” package and the custom function “DynNom_czx_lrm.” The non-invasive model was based on odds ratios (ORs) and *p*-values with 95% confidence intervals (CIs) derived from the MLR analysis. The model’s discrimination was evaluated by calculating the area under the curve (AUC) and C-statistic. Calibration was assessed using calibration curves created with 1,000 bootstrap samples. The CIC and DCA curves were used to evaluate the clinical applicability of the model across varying threshold probabilities (Figure 1). Statistical significance was defined as a *p-value of* < 0.05 (two-sided).

## Results

### Baseline characteristics

A total of 739 eligible subjects were included in the final cohort, of whom 488 (66.0%) were diagnosed with severe CAS. The cohort comprised 498 male participants (67.4%) and 241 female participants (32.6%). The baseline characteristics of the training and test sets are summarized in Table 1. No significant differences were observed between the two sets regarding baseline characteristics (all *p* > 0.05). The baseline characteristics of patients in the severe CAS and non-severe CAS groups are provided in Supplementary Table 1.

**Table 1:** Baseline characteristics of participants in different group.

Variables	Total (*n* = 739)	Test set (*n* = 225)	Train set (*n* = 514)	*p*
Age, Median (Q1, Q3)	65 (58, 70)	65 (57, 70)	66 (58, 71)	0.115
Sex, *n* (%)				0.509
Female	241 (33)	69 (31)	172 (33)	
Male	498 (67)	156 (69)	342 (67)	
Stroke, *n* (%)				0.571
No	166 (22)	54 (24)	112 (22)	
Yes	573 (78)	171 (76)	402 (78)	
Hypertension, *n* (%)				0.964
No	211 (29)	65 (29)	146 (28)	
Yes	528 (71)	160 (71)	368 (72)	
Diabetes, *n* (%)				0.988
No	534 (72)	162 (72)	372 (72)	
Yes	205 (28)	63 (28)	142 (28)	
Hyperuricemia, *n* (%)				0.235
No	539 (73)	157 (70)	382 (74)	
Yes	200 (27)	68 (30)	132 (26)	
Fg (g/L), Median (Q1, Q3)	3.33 (2.89, 3.95)	3.4 (2.9, 3.96)	3.29 (2.88, 3.93)	0.280
PT (s), Median (Q1, Q3)	12.9 (12.5, 13.5)	12.9 (12.5, 13.5)	12.9 (12.5, 13.4)	0.930
APTT (s), Median (Q1, Q3)	34.8 (30.6, 39)	34 (30.2, 39.1)	35.1 (30.72, 39)	0.167
TT (s), Median (Q1, Q3)	16.6 (15.9, 17.2)	16.5 (15.9, 17.2)	16.6 (15.9, 17.28)	0.856
INR, Median (Q1, Q3)	0.99 (0.94, 1.04)	0.98 (0.95, 1.05)	0.99 (0.94, 1.04)	0.753
HbAlc (%), Median (Q1, Q3)	6 (5.4, 6.8)	6 (5.3, 6.8)	6 (5.4, 6.8)	0.536
ALB (g/L), Median (Q1, Q3)	38.9 (36.55, 41)	39 (37, 41)	38.7 (36.4, 41)	0.584
TP (g/L), Median (Q1, Q3)	66.5 (62, 70.95)	66 (62.2, 70)	66.8 (62, 71)	0.256
DBIL (umol/L), Median (Q1, Q3)	3.5 (2.5, 4.6)	3.4 (2.4, 4.2)	3.6 (2.6, 4.6)	0.164
TBIL (umol/L), Median (Q1, Q3)	12.7 (9.9, 15.9)	12.6 (9.9, 16)	12.85 (9.83, 15.88)	0.987
ALT (U/L), Median (Q1, Q3)	18 (13, 25)	18 (13, 25)	18 (13, 25)	0.948
Na (mmol/L), Median (Q1, Q3)	141 (140, 143)	142 (140, 143)	141 (140, 143)	0.434
Cl (mmol/L), Median (Q1, Q3)	104 (102, 106)	104 (102, 106)	104 (102, 106)	0.581
RBP, Median (Q1, Q3)	41 (34, 49)	41 (35, 48)	41 (34, 49)	0.944
Mg (mmol/L), Median (Q1, Q3)	0.88 (0.81, 0.97)	0.87 (0.8, 0.96)	0.89 (0.81, 0.98)	0.133
PaCO_2_, Median (Q1, Q3)	26 (24.2, 28)	25.9 (24, 28)	26 (24.3, 28)	0.324
Ca (mmol/L), Median (Q1, Q3)	2.2 (2.13, 2.27)	2.2 (2.13, 2.26)	2.21 (2.13, 2.27)	0.566
K (mmol/L), Median (Q1, Q3)	3.84 (3.61, 4.09)	3.82 (3.58, 4.1)	3.84 (3.61, 4.08)	0.537
HCY, Median (Q1, Q3)	12.3 (10, 15.35)	12.3 (10, 15.6)	12.25 (10, 15.1)	0.523
P (mmol/L), Mean ± SD	1.08 ± 0.21	1.08 ± 0.22	1.09 ± 0.21	0.924
URCA (umol/L), Median (Q1, Q3)	360 (296, 421.5)	366 (302, 428)	356.5 (291, 420)	0.078
BUN (mmol/L), Median (Q1, Q3)	4.7 (3.8, 5.9)	4.66 (3.8, 5.83)	4.75 (3.89, 5.92)	0.755
CYC (mg/L), Median (Q1, Q3)	1.04 (0.88, 1.22)	1.04 (0.89, 1.22)	1.04 (0.88, 1.22)	0.630
β2MG (mg/L), Median (Q1, Q3)	2.05 (1.74, 2.47)	2.09 (1.75, 2.48)	2.03 (1.73, 2.47)	0.478
CREA (mmol/L), Median (Q1, Q3)	72.6 (61.9, 85.2)	74.6 (62.1, 88.8)	71.7 (61.4, 82.88)	0.115
Glucose (mmol/L), Median (Q1, Q3)	5.21 (4.69, 6.3)	5.21 (4.75, 6.13)	5.19 (4.68, 6.37)	0.849
PLT (10^9/L), Median (Q1, Q3)	241 (202, 290.5)	235 (197, 290) 24	2.5 (203.25, 290.75)	0.631
HDL (mmol/L), Median (Q1, Q3)	1.1 (0.94, 1.3)	1.09 (0.97, 1.3)	1.1 (0.93, 1.29)	0.871
LDL (mmol/L), Median (Q1, Q3)	2.82 (2.22, 3.58)	2.76 (2.17, 3.73)	2.84 (2.25, 3.55)	0.979
TG (mmol/L), Median (Q1, Q3)	1.47 (1.01, 2.17)	1.5 (1.14, 2.27)	1.46 (0.97, 2.15)	0.067
CHO (mmol/L), Median (Q1, Q3)	4.77 (3.99, 5.65)	4.82 (3.93, 5.79)	4.76 (4.05, 5.59)	0.632
hsCRP (mg/L), Median (Q1, Q3)	1.61 (0.66, 4.34)	1.61 (0.65, 4.88)	1.62 (0.66, 4.33)	0.651
CRP (mg/L), Median (Q1, Q3)	4.9 (4.9, 5.36)	4.9 (4.9, 6.85)	4.9 (4.9, 5.13)	0.307
RDWSD (fL), Median (Q1, Q3)	41.3 (39.1, 43.4)	41.4 (39.2, 43.7)	41.2 (39.1, 43.27)	0.403
HCT (%), Median (Q1, Q3)	40.5 (38, 43.1)	40.8 (38.4, 43)	40.4 (37.82, 43.1)	0.466
MCH (pg), Median (Q1, Q3)	30 (28.5, 31.3)	30 (28.8, 31.3)	29.9 (28.42, 31.3)	0.572
MONO (10^9/L), Median (Q1, Q3)	0.46 (0.36, 0.59)	0.47 (0.37, 0.6)	0.46 (0.35, 0.58)	0.658
MCV (fL), Median (Q1, Q3)	88.9 (84.65, 92.6)	89.1 (85.1, 92.8)	88.9 (84.6, 92.6)	0.447
LYM (10^9/L), Median (Q1, Q3)	1.81 (1.33, 2.35)	1.89 (1.38, 2.45)	1.77 (1.32, 2.33)	0.148
RBC (10^12/L), Median (Q1, Q3)	4.62 (4.28, 5.04)	4.6 (4.31, 5.03)	4.63 (4.26, 5.04)	0.994
HGB (g/L), Median (Q1, Q3)	136 (126, 146)	136 (128, 147)	136 (126, 146)	0.372
BASO (10^9/L), Median (Q1, Q3)	0.02 (0.01, 0.03)	0.02 (0, 0.03)	0.02 (0.01, 0.03)	0.719
MCHC (g/L), Median (Q1, Q3)	336 (329, 343)	337 (329, 343)	335 (328, 343)	0.547
RDWCV (%), Median (Q1, Q3)	12.9 (12.4, 13.5)	12.9 (12.4, 13.4)	12.9 (12.4, 13.6)	0.682
WBC (10^9/L), Median (Q1, Q3)	7.43 (6.05, 8.95)	7.18 (5.97, 8.94)	7.57 (6.11, 8.98)	0.28
BASO_A (%), Median (Q1, Q3)	0.2 (0.1, 0.4)	0.2 (0.04, 0.4)	0.2 (0.1, 0.4)	0.925
MONO_A (%), Median (Q1, Q3)	6.3 (5.2, 7.6)	6.5 (5.4, 7.7)	6.2 (5.2, 7.6)	0.177
Stenosis, *n* (%)				0.855
No	251 (34)	78 (35)	173 (34)	
Yes	488 (66)	147 (65)	341 (66)	

### Selected predictor in the train set

**Figure 2:**
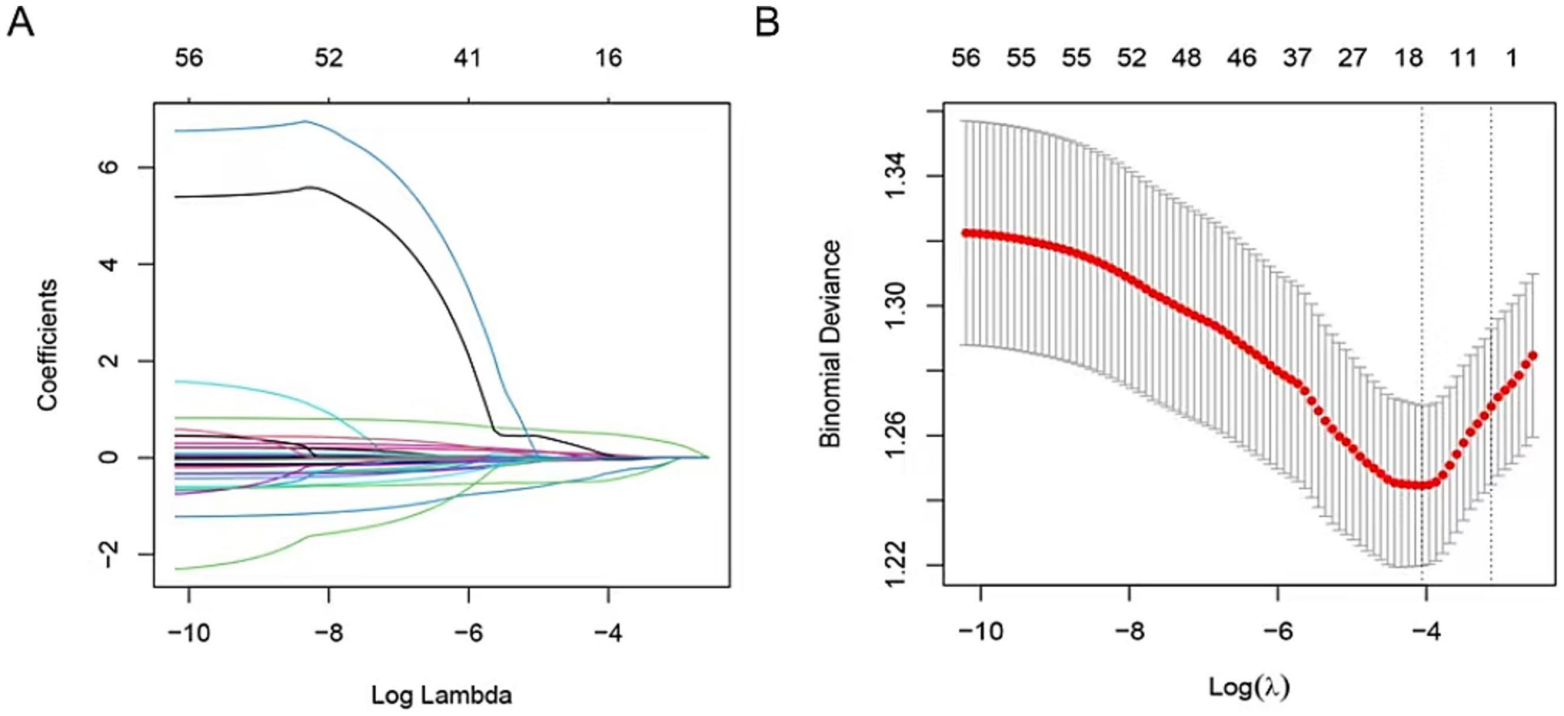
The predictors’ selection by LASSO method. **(A)** Coefficients of all predictors gradually returned to zeros by using 10-fold cross-validation. **(B)** Coefficients of 11 predictors were non-zero at the leftmost dashed line (*λ* = lambda. min). Min, minimum.

To identify predictive factors for severe CAS in IS patients, the LASSO regularization method was applied to the data of 523 patients in the training set. This process yielded 11 potential predictors after shrinkage at the optimal lambda value (*λ* = 0.030205). Some regression coefficients were reduced to zero (Figures 2A,B), including stroke history, total protein (TP), serum sodium (Na), homocysteine (HCY), creatinine, high-density lipoprotein (HDL), cholesterol (CHO), high-sensitivity C-reactive protein (hsCRP), C-reactive protein (CRP), mean corpuscular hemoglobin concentration (MCHC), and basophil percentage (BASO_A). The predictors identified through LASSO analysis were further subjected to multivariate logistic regression (MLR) to confirm independent risk factors for severe CAS.

### Variable multicollinear analysis

To ensure the reliability of the multivariate model, variable collinearity was assessed. Correlation coefficients between variables were analyzed (Supplementary Table 2), along with variance inflation factor (VIF) and tolerance values (Table 2). The results indicated no multicollinearity among variables, as all correlation coefficients were below 0.8, VIF values were under 5, and tolerance values exceeded 0.1.

**Table 2:** The result of the VIF and Tolerance between the variables in the MLR model.

Term	VIF	Tolerance
stroke	1.02	0.98
BASO.A	1.04	0.96
Na	1.12	0.89
hsCRP	1.27	0.78
CRP	1.24	0.8
MCHC	1.11	0.9

### Model development

**Figure 3:**
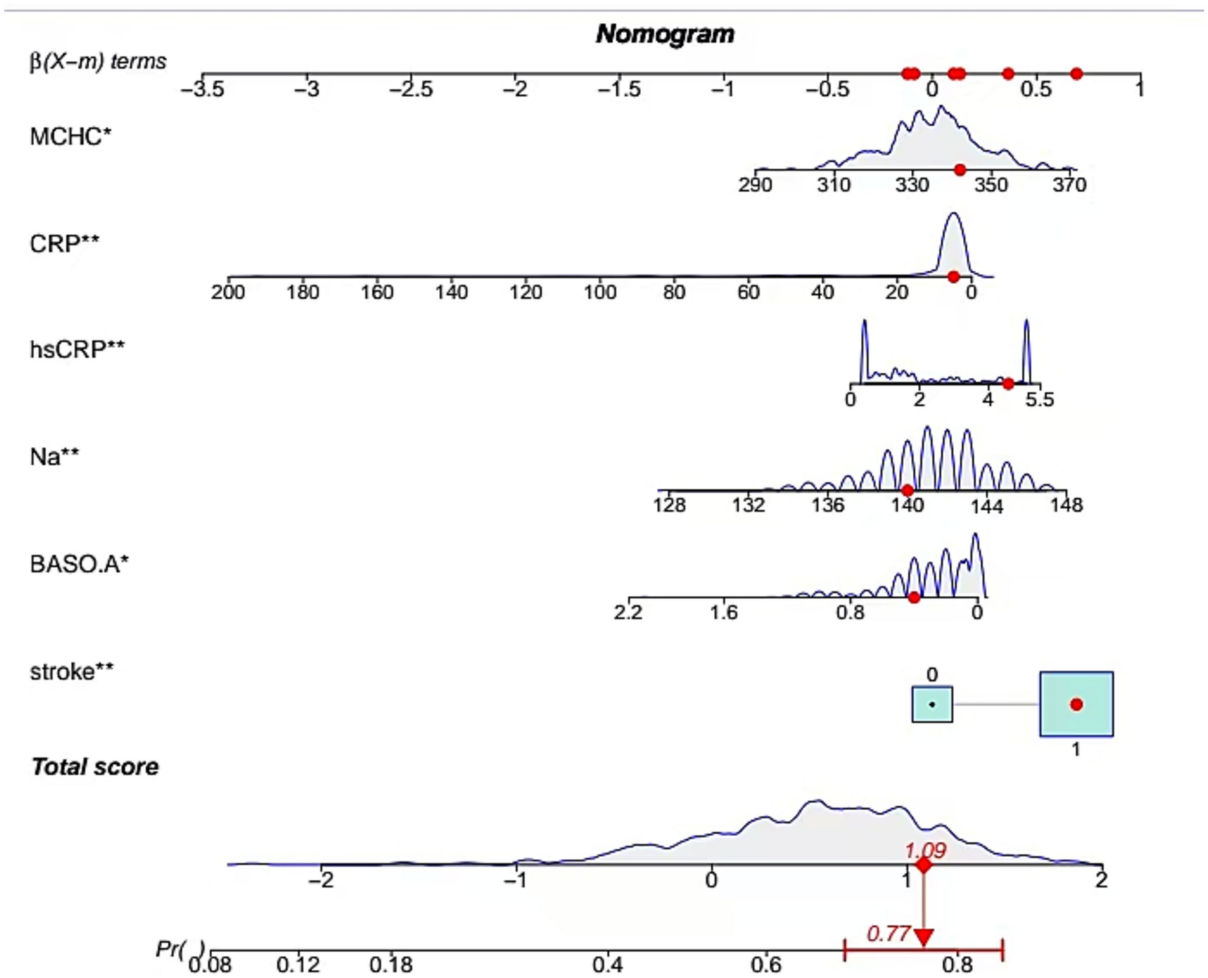
Development of nomogram. A case of the nomogram model showed that the probability of severe CAS was 0.77.

Through MLR analysis, six variables—stroke history, serum sodium (Na), hsCRP, CRP, MCHC, and BASO_A—were identified as significant predictors of severe CAS (Table 3). These variables were incorporated into the final prediction model. A non-invasive predictive model integrating these six variables was developed and visualized. Figure 3 illustrates a case where a patient with a history of stroke and specific biomarker values (BASO.A = 0.4, Na = 141 mmol/L, hsCRP = 4.3 mg/L, CRP = 6 mg/L, MCHC = 341 g/L) was assessed using a web-based dynamic nomogram tool. This tool allows clinicians to input patient-specific parameters and obtain individualized risk predictions based on their estimated effect sizes *β*(X − m). Each biomarker contributes to the total risk score, which is calculated by summing the individual point values from the nomogram. The red dots indicate the patient’s exact values, and the computed total score is approximately 1.09, corresponding to a predicted probability of 77%, suggesting a high risk of the condition. The probability distribution curve (gray area) provides context within the dataset, placing this patient in the higher-risk category. This tool enhances clinical decision-making by enabling early risk assessment and supporting further diagnostic evaluations and personalized interventions. Weighted scores were assigned to each variable in the model. The predicted probability of severe CAS ranged from 0.2 to 0.9.

**Table 3:** The result of multivariable logistic regression analysis.

	Coef	S.E.	Wald Z	OR	Lower 0.95	Upper 0.95	*p*
Stroke = 1	0.4992	0.2347	2.13	0.6070	0.3832	0.9616	0.0334
TP	−0.0224	0.0159	−1.41	0.8176	0.6181	1.0815	0.1582
Na	0.1169	0.0377	3.10	1.4199	1.1378	1.7721	0.0019
HCY	0.0087	0.0169	0.51	1.0453	0.8828	1.2378	0.6073
Creatinine	0.0056	0.0050	1.12	1.1279	0.9140	1.3918	0.2620
HDL	−0.4285	0.3397	−1.26	0.8571	0.6744	1.0892	0.2072
CHO	−0.0561	0.0854	−0.66	0.9175	0.7096	1.1863	0.5111
hsCRP	0.2238	0.0624	3.59	2.2706	1.4505	3.5546	0.0003
CRP	−0.0157	0.0053	−2.97	0.9964	0.9940	0.9987	0.0030
MCHC	0.018	0.0084	2.15	1.3098	1.0236	1.6760	0.0319
BASO_A	−0.7121	0.3561	−2.00	0.8077	0.6551	0.9957	0.0455

### Validation of model

**Figure 4:**
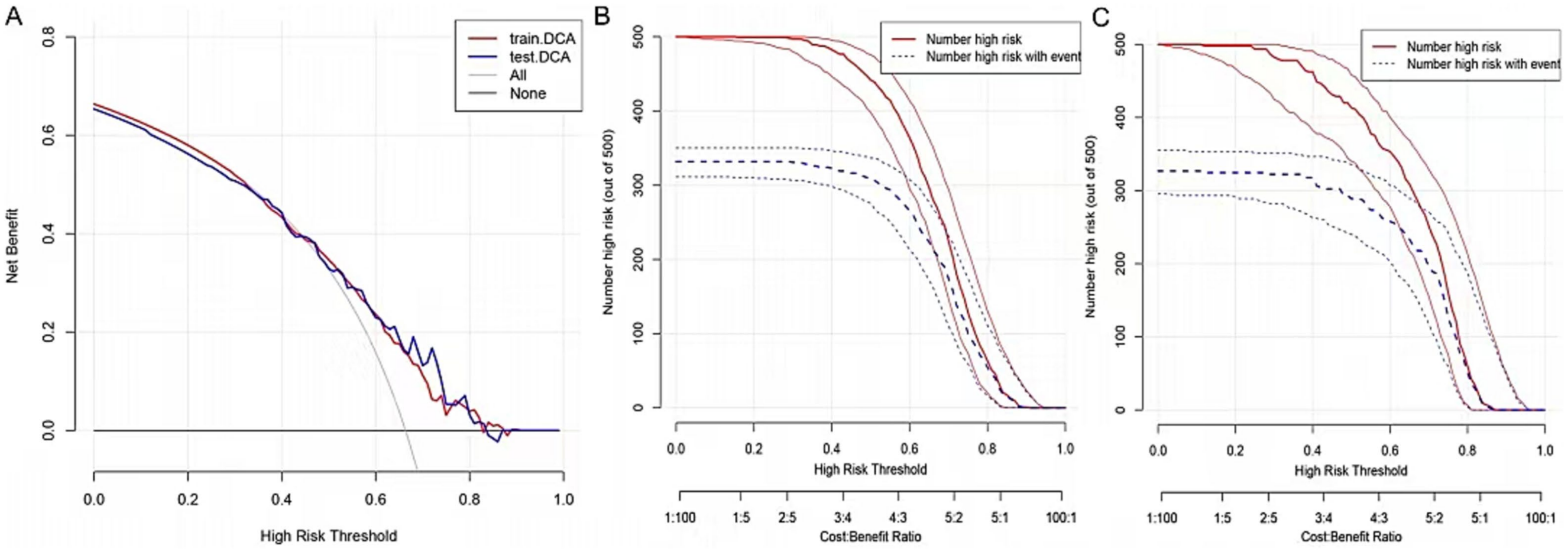
Validation of nomogram. **(A)** The DCA demonstrated the net benefit and threshold probability of nomogram in the train set and test set. **(B)** The CIC in the train set. **(C)** The CIC in the test set.

**Figure 5:**
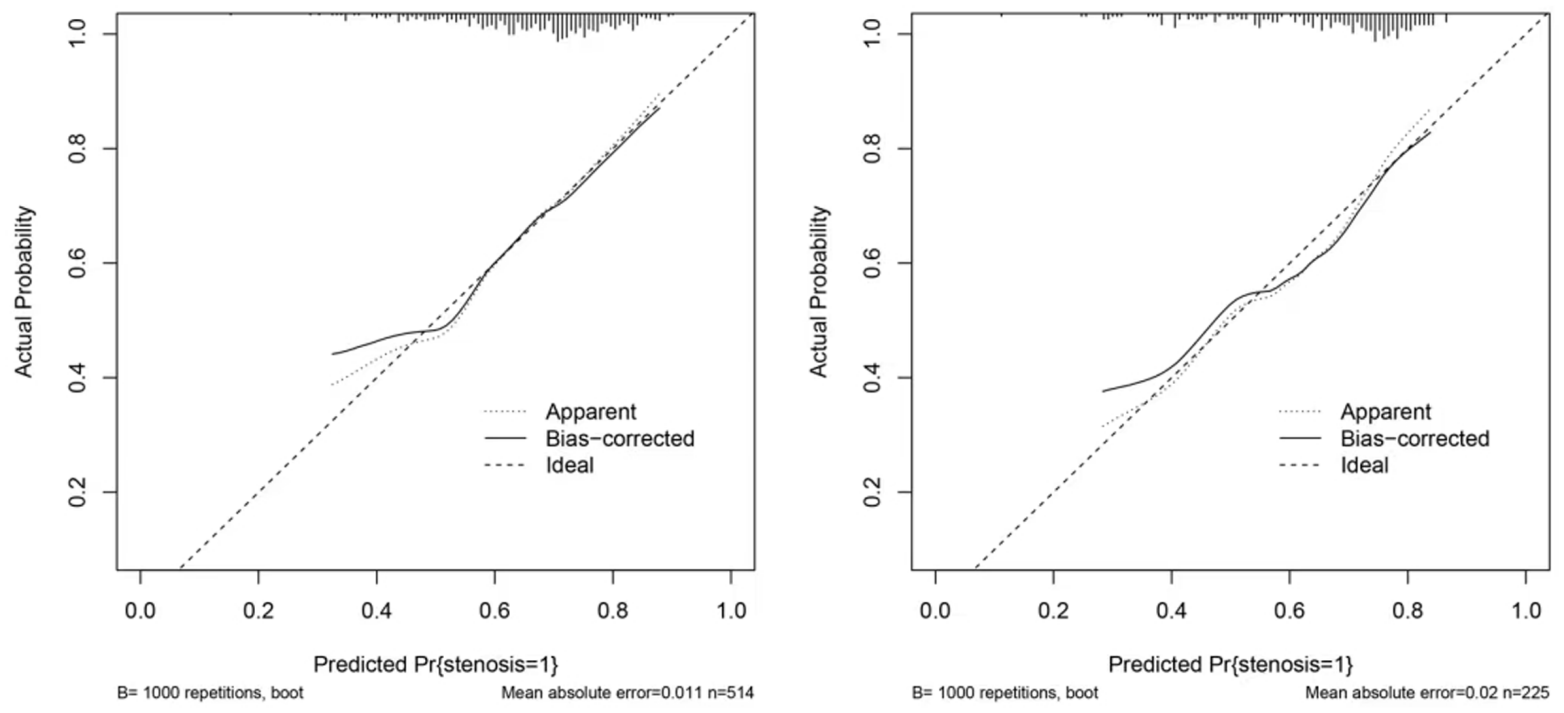
Calibration of nomogram. **(A)** The calibration curve in the train set. **(B)** The calibration curve in the test set. The calibration curve in both the train and test sets did not cross the diagonal bisector line, suggesting that the prediction models had a strong concordance performance in both groups; this indicates the model performed well in both groups.

**Figure 6:**
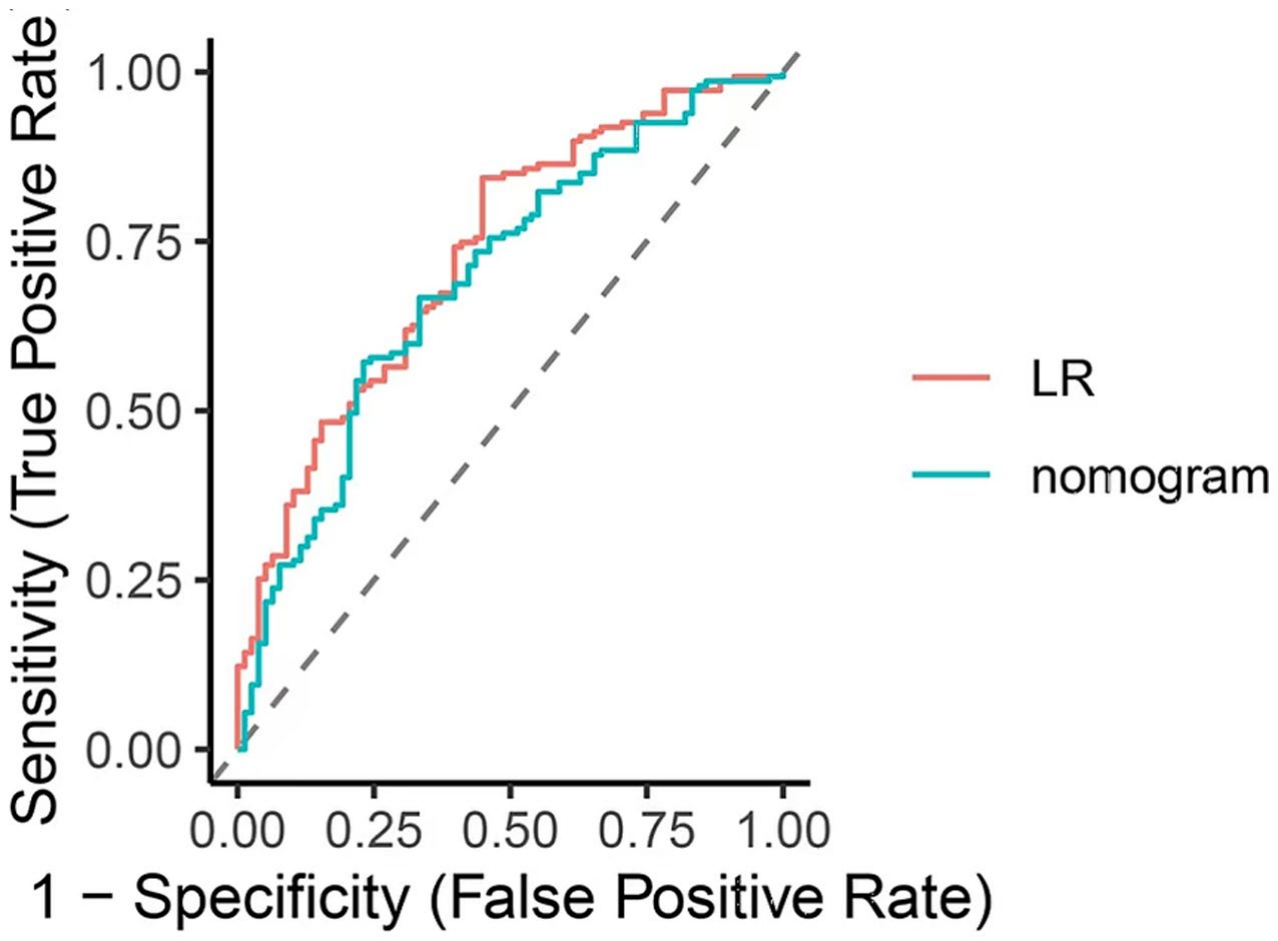
ROC plot for comparing nomogram with LR model in the test set (LR model: multivariable logistic regression model comprising 11 variables; nomogram model: multivariable logistic regression model comprising 6 variables).

The clinical utility of the predictive model was assessed using decision curve analysis (DCA) and clinical impact curves (CICs). DCA demonstrated net benefit and threshold probabilities for both the training and test sets (Figure 4A). CICs further supported the model’s clinical usefulness (Figures 4B,C). Calibration plots indicated that the model achieved a good fit for predicting severe CAS in both the training and test sets (Figures 5A,B). The discrimination ability of the model was evaluated using receiver operating characteristic (ROC) curves (Figure 6). Two models had above 0.7 AUC values. The values of C-statistic/AUC of the LR model and nomogram model in the test set were 0.73 and 0.70, respectively.

### Application of the web-based dynamic model

The web-based dynamic model was applied to predict outcomes for individual cases. Nine individual cases were used to demonstrate the predictive performance of the model. The model’s prediction results varied across different cases, and the prediction probabilities and confidence intervals are shown in Supplementary Table 3. Remarkably, the predicted outcomes were consistent with the actual prognoses of all patients, demonstrating the model’s robust predictive accuracy.

## Discussion

To the best of our knowledge, this study represents the first attempt to develop a dynamic nomogram for predicting severe carotid artery stenosis (CAS) in Chinese patients with ischemic stroke (IS). This web-based non-invasive model provided an optimal prediction of severe CAS in IS patients through internal validation. One of the greatest advantages of this study was that the non-invasive model based on available factors could provide patient-specific, evidence-based advice for clinicians, which benefited patients who did not receive or were not suitable for imaging examinations. Our prediction model integrates six significant predictors: history of stroke, serum sodium, hypersensitive C-reactive protein (hsCRP), C-reactive protein (CRP), basophil percentage, and mean corpuscular hemoglobin concentration (MCHC). Importantly, multicollinearity was not observed among these variables, as confirmed by variable correlation plots, variance inflation factor (VIF) values, and tolerance values. Internal validation demonstrated the model’s good discrimination (C-statistic = 0.70) and satisfactory calibration. Decision curve analysis (DCA) and clinical impact curves (CICs) confirmed the clinical utility of the model, indicating that IS patients could benefit from clinical decisions informed by this non-invasive tool. Moreover, the calibration plot revealed strong agreement between predicted and actual probabilities, while the web-based individualized prediction model exhibited robust predictive performance.

According to Fei Han et al. (2), in a prospective study involving 1,082 stroke-free participants, 34 individuals experienced an ischemic stroke during an average follow-up of 4.2 years, with arterial atherosclerotic stenosis identified as a significant risk factor for future IS among asymptomatic individuals. Similarly, a study among 740 Japanese male participants (average age: 68 years) reported prevalence rates of 20.7% for mild and 4.5% for severe intracranial atherosclerotic stenosis (ICAS) (10). In a Chinese observational cohort study, 44.2% of young IS patients were categorized into the stenosis group (middle cerebral artery [MCA] stenosis ≥50%), while 55.8% were placed in the no stenosis group (MCA stenosis <50% or no stenosis) (11). In comparison, our study identified 488 severe CAS cases (66.0%), a proportion notably higher than the 44.2% reported. The severity of stenosis also exacerbates IS symptoms. For instance, IS patients with atherosclerotic MCA stenosis exhibited higher plaque burdens in the symptomatic group compared to the asymptomatic group. Moderate-to-severe stenosis was more prevalent, underscoring the association between stenosis severity and IS symptomatology (12). Furthermore, severe intracranial large vessel stenosis contributes to stroke-related complications. Hilal et al. (13) reported that intracranial stenosis, as defined by magnetic resonance angiography (MRA), could impair cognitive function due to reduced cerebral blood flow from atherosclerotic stenosis. In a prospective cohort study of 200 patients aged >40 years diagnosed with MCA stenosis via transcranial Doppler (TCD), 3.8% developed IS or transient ischemic attack (TIA) during follow-up (14). Severe CAS has also been independently associated with ipsilateral acute IS (15) and an increased 90-day IS risk in TIA patients (*p* < 0.01) (16). In addition, a strong correlation has been observed between cerebral artery stenosis and IS occurrence both before and after revascularization (*p* < 0.01) (7).

This study builds on previous research by providing a novel web-based dynamic nomogram to predict severe carotid artery stenosis (CAS) in patients with ischemic stroke (IS). Consistent with earlier findings, our study found that metabolic syndrome, such as sodium ion disorder, is associated with severe CAS (17). However, our findings regarding high-density lipoprotein cholesterol (HDL-C) differed from those of previous studies. A study (18) comprised 194 intracranial atherosclerotic stenosis and found that lower HDL-C was associated with the presence of arterial stenosis (*p* < 0.001), which was conversely with this study. A total of 412 patients (35–93 years old) with ischemic stroke were more prevalent in the MCA stenosis group; interestingly, there was no significant difference in HCY levels between the MCA stenosis and no stenosis groups at baseline and matched for age and sex (19). Consistently with it, the level of HCY was not an independent risk of severe CAS in this study. In a Japan study (20), 8 out of 103 patients with type II diabetes developed arterial stenosis, and glucose fluctuation was significantly higher in the severe stenosis group (≥70%) than in the non-severe stenosis group, instead of mean blood glucose and HbA1c, which was conversely to our study that random glucose was not an independent risk factor. Conventional risk factors, including age, a history of hyperhomocysteinemia, hypertension, diabetes mellitus, TG, HDL, LDL, and dyslipidemia, were excluded from the model in this study (21). The history of stroke was a risk factor for progressive posterior cerebral artery stenosis after revascularization (7). However, our finding that MCHC was a risk factor for severe CAS in IS patients had not been previously reported. There had been only one report that the group administered with cholesterol plus HDMC (high dose *M. charantia*) showed significantly lower MCHC than that of the control group in cholesterol-fed experimental rats before (22). Sun et al. (23) found that the higher the carotid stenosis rate, the higher the level of hsCRP in the ulcerated plaque group in the cerebral artery stenosis patients with IS, which was consistent with our study. Li et al. (24) found that the level of CRP was not an independent risk factor in cerebral artery stenosis patients with IS, which was in converse to our study. There were also significant associations between the percentages of basophil and acute ischemic cerebrovascular events at 3-month and 1-year follow-up in patients (25), which was consistent to our study. Furthermore, the model included factors that were easily acquired in this study so the model could be easily applied in clinical practice. Serum sodium levels are indicative of the body’s fluid and electrolyte balance. Hyponatremia (low serum sodium) has been associated with increased morbidity and mortality in various conditions, including cardiovascular diseases. Studies suggest that hyponatremia may exacerbate atherosclerosis by promoting endothelial dysfunction and inflammation, thereby increasing the risk of CAS (26). Conversely, hypernatremia (high serum sodium) can lead to increased blood viscosity and hypertension, both of which are risk factors for atherosclerosis and CAS (27). Basophils are a type of white blood cell involved in immune responses and inflammation. An elevated basophil percentage may reflect an ongoing inflammatory process. Chronic inflammation is a well-known contributor to atherosclerosis, which can lead to the development and progression of CAS. Therefore, an increased basophil percentage might be associated with a higher risk of carotid artery stenosis. In men, a low basophil-to-WBC ratio was linked to greater plaque instability, suggesting a protective role of basophils. In contrast, in women, a high basophil-to-WBC ratio was associated with increased plaque instability, hemorrhage, and thrombosis, highlighting a sex-specific dual role of basophils in vascular pathology (28). This highlights the dual nature of basophils in modulating inflammatory responses and their critical involvement in plaque progression, neovascularization, and thrombotic events.

The establishment of the prediction model for patients has always been a hot topic (29). In this study, we enrolled 739 IS patients to develop and validate the non-invasive web-based model based on independent risk factors. Six variables were considered as risk factors of severe CAS in this study, including the history of stroke, Na, hsCRP, CRP, BASO_A, and MCHC. Considering the fact that all variables came from clinical variables that were routinely collected, this dynamic model could easily be applied to IS patients to improve clinical decision-making. The wed-based model displayed accurate predictive power through internal validation in this study. The clinical applicability of this dynamic nomogram is enhanced by its use of routine clinical variables, allowing for widespread implementation. Internal validation demonstrated its predictive accuracy, with an AUC of 0.70 in the test set, indicating acceptable discrimination. By incorporating this model into clinical practice, neurologists can stratify patients by risk and adopt appropriate interventions, particularly for those unable to undergo imaging studies. Despite the strengths of imaging modalities such as MRA or TCD for detecting atherosclerotic stenosis (13, 14), our non-invasive model offers a practical and accessible alternative.

The dynamic nomogram developed in this study offers a novel approach to individualized risk assessment for severe carotid artery stenosis (CAS) in ischemic stroke (IS) patients. Health professionals can utilize the nomogram to implement targeted interventions based on the calculated risk scores of various predictors for each patient, thereby enhancing the efficiency and precision of clinical interventions. However, using a traditional nomogram may pose challenges for non-professional statisticians due to its requirement for manual risk calculations across multiple variables.

To address this limitation, we developed a web-based dynamic nomogram^1^ based on the prediction model. This online tool allows clinicians to input patient-specific predictors and instantly obtain an individualized CAS probability with a 95% confidence interval. The web-based nomogram significantly simplifies the application process, facilitates real-time decision-making, and enables the development of tailored, risk-based, and time-sensitive treatment strategies. Moreover, it provides a convenient resource for IS patients and their caregivers to implement personalized interventions based on the nomogram results.

### Limitations

This study had several limitations. First, the data were derived from a single-center retrospective study. Future research incorporating data from multiple medical centers and prospective cohort designs is necessary to validate and generalize our findings.

Second, this study only included random glucose measurements rather than glycated hemoglobin (HbA1c) as not all patients underwent HbA1c testing. Further research, including HbA1c, may provide more comprehensive insights into the relationship between glucose metabolism and CAS.

## Conclusion

In this study, six clinical variables—stroke history, serum sodium (Na), hypersensitive C-reactive protein (hsCRP), C-reactive protein (CRP), basophil percentage (BASO_A), and mean corpuscular hemoglobin concentration (MCHC)—were identified as potential predictors for severe CAS. A non-invasive prediction model incorporating these variables was successfully developed.

The web-based dynamic nomogram derived from this model allows healthcare professionals to easily identify IS patients at high risk of severe CAS, especially those unable to undergo imaging examinations. This tool facilitates the implementation of individualized, risk-based, and time-sensitive treatment strategies, which may ultimately improve patient outcomes and optimize resource utilization.

## Data Availability

The original contributions presented in the study are included in the article/Supplementary material, further inquiries can be directed to the corresponding author.

## Footnotes

^1^Accessible at: https://huangjian.shinyapps.io/DynNomapp/.
