# Supplementary figures and images for: Development and validation of a web-based dynamic nomogram to predict individualized risk of severe carotid artery stenosis based on digital subtract angiography
Source: Front Neurol. 2025 Mar 24;16:1565395. doi: 10.3389/fneur.2025.1565395 (PMC11973061; doi:10.3389/fneur.2025.1565395)

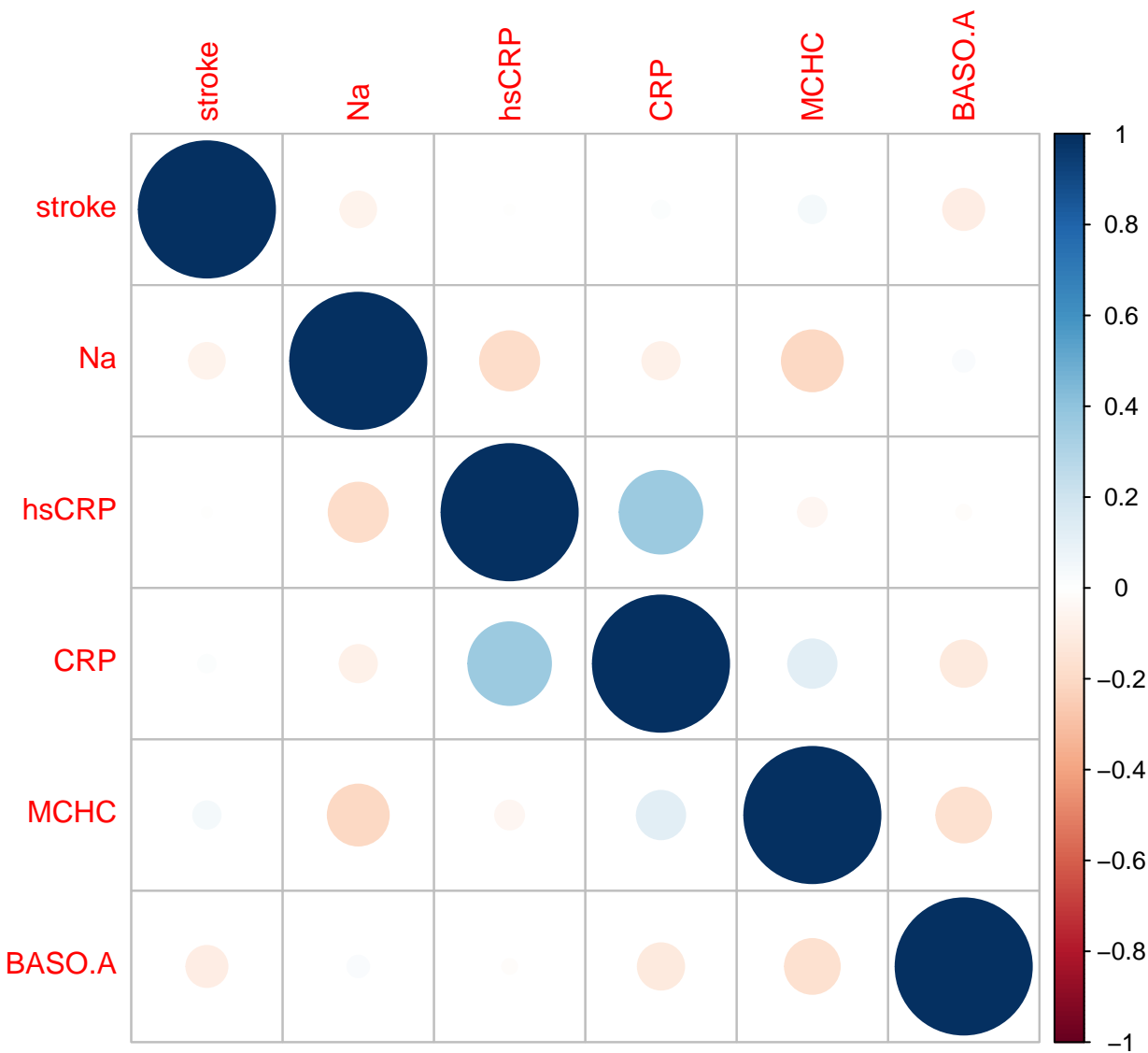

Supplement: Supplementary file 1 [file Data_Sheet_1.pdf]
