# Supplementary material for: Development and validation of a web-based dynamic nomogram to predict individualized risk of severe carotid artery stenosis based on digital subtract angiography
Source: Front Neurol. 2025 Mar 24;16:1565395. doi: 10.3389/fneur.2025.1565395 (PMC11973061; doi:10.3389/fneur.2025.1565395)
Supplement: Supplementary file 2 [file Table_1.docx]

**Supplement Table 1** Baseline characteristics of participants in non-severe LASS group and severe LAAS group

| Variables | Total (n = 739) | non-severe LAAS (n = 251) | severe LAAS  (n = 488) | p |
| --- | --- | --- | --- | --- |
| age, Median (Q1,Q3) | 65 (58, 70) | 65 (57, 70) | 66 (58, 70) | 0.517 |
| sex, n (%) |  |  |  | 0.024 |
| Female | 241 (33) | 96 (38) | 145 (30) |  |
| male | 498 (67) | 155 (62) | 343 (70) |  |
| stroke, n (%) |  |  |  | 0.001 |
| no | 166 (22) | 80 (32) | 86 (18) |  |
| yes | 573 (78) | 171 (68) | 402 (82) |  |
| hypertension, n (%) |  |  |  | 0.316 |
| no | 211 (29) | 78 (31) | 133 (27) |  |
| yes | 528 (71) | 173 (69) | 355 (73) |  |
| diabetes, n (%) |  |  |  | 0.587 |
| no | 534 (72) | 185 (74) | 349 (72) |  |
| yes | 205 (28) | 66 (26) | 139 (28) |  |
| hyperuricemia, n (%) |  |  |  | 0.784 |
| no | 539 (73) | 181 (72) | 358 (73) |  |
| yes | 200 (27) | 70 (28) | 130 (27) |  |
| Fg, Median (Q1,Q3) | 3.33 (2.89, 3.95) | 3.36 (2.9, 3.83) | 3.3 (2.89, 4) | 0.839 |
| PT, Median (Q1,Q3) | 12.9 (12.5, 13.5) | 12.8 (12.4, 13.4) | 12.95 (12.5, 13.5) | 0.043 |
| APTT, Median (Q1,Q3) | 34.8 (30.6, 39) | 35.5 (31.1, 38.9) | 34.2 (30.2, 39.1) | 0.069 |
| TT, Median (Q1,Q3) | 16.6 (15.9, 17.2) | 16.6 (16, 17.2) | 16.5 (15.8, 17.3) | 0.455 |
| INR, Median (Q1,Q3) | 0.99 (0.94, 1.04) | 0.98 (0.94, 1.03) | 0.99 (0.95, 1.04) | 0.036 |
| HbAlc, Median (Q1,Q3) | 6 (5.4, 6.8) | 6.1 (5.4, 6.9) | 5.9 (5.4, 6.8) | 0.215 |
| ALB, Median (Q1,Q3) | 38.9 (36.55, 41) | 39 (36.6, 41.6) | 38.45 (36.5, 41) | 0.182 |
| TP, Median (Q1,Q3) | 66.5 (62, 70.95) | 67.3 (62.3, 72) | 65.95 (62, 70) | 0.035 |
| DBIL, Median (Q1,Q3) | 3.5 (2.5, 4.6) | 3.6 (2.65, 4.55) | 3.5 (2.4, 4.6) | 0.571 |
| TBIL, Median (Q1,Q3) | 12.7 (9.9, 15.9) | 13 (10.05, 16.05) | 12.5 (9.8, 15.6) | 0.172 |
| ALT, Median (Q1,Q3) | 18 (13, 25) | 17 (13, 24.5) | 19 (13, 26) | 0.235 |
| Na, Median (Q1,Q3) | 141 (140, 143) | 141 (139, 143) | 142 (140, 143) | 0.028 |
| Cl, Median (Q1,Q3) | 104 (102, 106) | 104 (102, 106) | 104 (102, 106) | 0.638 |
| RBP, Median (Q1,Q3) | 41 (34, 49) | 41 (34, 48) | 42 (34.75, 49) | 0.612 |
| Mg, Median (Q1,Q3) | 0.88 (0.81, 0.97) | 0.87 (0.81, 0.98) | 0.88 (0.81, 0.97) | 0.967 |
| PaCO_2_, Median (Q1,Q3) | 26 (24.2, 28) | 26 (24, 28) | 26 (24.3, 28) | 0.865 |
| CA, Median (Q1,Q3) | 2.2 (2.13, 2.27) | 2.2 (2.14, 2.26) | 2.21 (2.13, 2.28) | 1 |
| K, Median (Q1,Q3) | 3.84 (3.61, 4.09) | 3.86 (3.6, 4.05) | 3.82 (3.61, 4.1) | 0.706 |
| HCY, Median (Q1,Q3) | 12.3 (10, 15.35) | 11.8 (9.8, 14.05) | 12.8 (10, 16.22) | 0.002 |
| P, Mean ± SD | 1.08 ± 0.21 | 1.1 ± 0.21 | 1.08 ± 0.21 | 0.123 |
| URCA, Median (Q1,Q3) | 360 (296, 421.5) | 347 (295.5, 421) | 363 (296, 422) | 0.496 |
| BUN, Median (Q1,Q3) | 4.7 (3.8, 5.9) | 4.5 (3.77, 5.69) | 4.8 (3.85, 6) | 0.098 |
| CYC, Median (Q1,Q3) | 1.04 (0.88, 1.22) | 1 (0.86, 1.16) | 1.05 (0.9, 1.26) | 0.026 |
| β2MG, Median (Q1,Q3) | 2.05 (1.74, 2.47） | 2 (1.68, 2.38） | 2.1 (1.77, 2.49) | 0.015 |
| Creatinine, Median (Q1,Q3) | 72.6 (61.9, 85.2) | 69.7 (60, 80.9) | 74.7 (62.9, 86.4) | 0.003 |
| GLU, Median (Q1,Q3) | 5.21 (4.69, 6.3) | 5.2 (4.69, 6.28) | 5.21 (4.7, 6.3) | 0.939 |
| PLT, Median (Q1,Q3) | 241 (202, 290.5) | 241 (205, 291.5) | 239.5 (201, 290) | 0.613 |
| HDL, Median (Q1,Q3) | 1.1 (0.94, 1.3) | 1.14 (0.96, 1.39) | 1.09 (0.94, 1.25) | 0.009 |
| LDL, Median (Q1,Q3) | 2.82 (2.22, 3.58) | 2.92 (2.29, 3.6) | 2.73 (2.19, 3.55) | 0.035 |
| TG, Median (Q1,Q3) | 1.47 (1.01, 2.17) | 1.47 (1, 2.29) | 1.48 (1.01, 2.11) | 0.794 |
| CHO, Median (Q1,Q3) | 4.77 (3.99, 5.65) | 4.98 (4.18, 5.69) | 4.67 (3.92, 5.59) | 0.017 |
| hsCRP, Median (Q1,Q3) | 1.61 (0.66, 4.34) | 1.35 (0.4, 3.95) | 1.8 (0.73, 4.35) | 0.009 |
| CRP, Median (Q1,Q3) | 4.9 (4.9, 5.36) | 4.9 (4.9, 4.97) | 4.9 (4.9, 5.53) | 0.341 |
| RDWSD, Median (Q1,Q3) | 41.3 (39.1, 43.4) | 41.1 (39.05, 43.1) | 41.35 (39.3, 43.6) | 0.105 |
| HCT, Median (Q1,Q3) | 40.5 (38, 43.1) | 40.5 (38.05, 42.95) | 40.5 (37.9, 43.3) | 0.981 |
| MCH, Median (Q1,Q3) | 30 (28.5, 31.3) | 29.9 (28.4, 31.1) | 30 (28.67, 31.4) | 0.202 |
| MONO, Median (Q1,Q3) | 0.46 (0.36, 0.59) | 0.46 (0.36, 0.55) | 0.47 (0.36, 0.6) | 0.079 |
| MCV, Median (Q1,Q3) | 88.9 (84.65, 92.6) | 88.9 (84.45, 92.55) | 89 (84.77, 92.6) | 0.447 |
| LYM, Median (Q1,Q3) | 1.81 (1.33, 2.35) | 1.85 (1.4, 2.42) | 1.77 (1.31, 2.35) | 0.191 |
| RBC, Median (Q1,Q3) | 4.62 (4.28, 5.04) | 4.62 (4.3, 5.04) | 4.62 (4.26, 5.03) | 0.384 |
| HGB, Median (Q1,Q3) | 136 (126, 146) | 136 (126, 145) | 136 (126, 147) | 0.495 |
| BASO, Median (Q1,Q3) | 0.02 (0.01, 0.03) | 0.02 (0.01, 0.03) | 0.01 (0, 0.03) | 0.011 |
| MCHC, Median (Q1,Q3) | 336 (329, 343) | 335 (327, 341) | 336 (329, 343) | 0.033 |
| RDWCV, Median (Q1,Q3) | 12.9 (12.4, 13.5) | 12.9 (12.4, 13.6) | 12.9 (12.4, 13.5) | 0.960 |
| WBC, Median (Q1,Q3) | 7.43 (6.05, 8.95) | 7.29 (5.97, 8.87) | 7.52 (6.11, 8.95) | 0.274 |
| BASO_A, Median (Q1,Q3) | 0.2 (0.1, 0.4) | 0.3 (0.1, 0.5) | 0.2 (0.04, 0.4) | 0.003 |
| MONO_A, Median (Q1,Q3) | 6.3 (5.2, 7.6) | 6.3 (5.3, 7.45) | 6.25 (5.2, 7.8) | 0.477 |

**Supplement Table 2** The result of magnitude of the correlation coefficients between the variables

|  | stroke | Na | hsCRP | CRP | MCHC | BASO.A |
| --- | --- | --- | --- | --- | --- | --- |
| stroke | 1 | -0.06910101 | -0.00498936 | 0.01655117 | 0.04042506 | -0.0911834 |
| Na | -0.06910101 | 1 | -0.18873996 | -0.07495953 | -0.20042296 | 0.02512303 |
| hsCRP | -0.00498936 | -0.18873996 | 1 | 0.36873464 | -0.0449512 | -0.01184897 |
| CRP | 0.01655117 | -0.07495953 | 0.36873464 | 1 | 0.12633535 | -0.11573553 |
| MCHC | 0.04042506 | -0.20042296 | -0.0449512 | 0.12633535 | 1 | -0.1621965 |
| BASO.A | -0.0911834 | 0.02512303 | -0.01184897 | -0.11573553 | -0.1621965 | 1 |

**Supplement Table 3** The predictive result of the nine case by web-based model

| stroke | BASO.A | Na | hsCRP | CRP | MCHC | Prediction | Lower.bound | Upper.bound |
| --- | --- | --- | --- | --- | --- | --- | --- | --- |
| 0 | 1 | 141 | 2 | 11 | 336 | 0.291 | 0.147 | 0.494 |
| 0 | 1 | 139 | 3 | 35 | 322 | 0.183 | 0.072 | 0.392 |
| 0 | 2 | 143 | 3 | 70 | 348 | 0.091 | 0.011 | 0.467 |
| 0 | 1 | 141 | 3 | 101 | 341 | 0.117 | 0.022 | 0.437 |
| 0 | 1 | 140 | 4 | 74 | 317 | 0.12 | 0.03 | 0.373 |
| 0 | 3 | 143 | 3 | 38 | 342 | 0.052 | 0.003 | 0.49 |
| 0 | 1 | 145 | 2 | 77 | 316 | 0.124 | 0.028 | 0.412 |
| 1 | 2 | 143 | 3 | 130 | 329 | 0.083 | 0.006 | 0.57 |
| 1 | 3 | 144 | 4 | 161 | 343 | 0.031 | 0.001 | 0.593 |
| 1 | 3 | 136 | 3 | 112 | 349 | 0.04 | 0.001 | 0.548 |
| 1 | 2 | 139 | 2 | 147 | 341 | 0.058 | 0.003 | 0.54 |
